# Identification of genes coding for putative wax ester synthase/diacylglycerol acyltransferase enzymes in terrestrial and marine environments

**DOI:** 10.1186/s13568-015-0128-1

**Published:** 2015-07-31

**Authors:** Mariana P Lanfranconi, Adrián F Alvarez, Héctor M Alvarez

**Affiliations:** Centro Regional de Investigación y Desarrollo Científico Tecnológico (CRIDECIT), Facultad de Ciencias Naturales, Universidad Nacional de la Patagonia San Juan Bosco y CIT-CHUBUT CONICET, Km 4-Ciudad Universitaria, 9000 Comodoro Rivadavia, Chubut Argentina; Departamento de Genética Molecular, Instituto de Fisiología Celular, Universidad Nacional Autónoma de México, 04510 Mexico City, Mexico

**Keywords:** Neutral lipids, WS/DGAT enzymes, Environmental diversity, Coastal marine sediments, Polluted arid soils

## Abstract

Synthesis of neutral lipids such as triacylglycerols (TAG) and wax esters (WE) is catalyzed in bacteria by wax ester synthase/diacylglycerol acyltransferase enzymes (WS/DGAT). We investigated the diversity of genes encoding this enzyme in contrasting natural environments from Patagonia (Argentina). The content of petroleum hydrocarbons in samples collected from oil-producing areas was measured. PCR-based analysis covered WS/DGAT occurrence in marine sediments and soil. No product was obtained in seawater samples. All clones retrieved from marine sediments affiliated with gammaproteobacterial sequences and within them, most phylotypes formed a unique cluster related to putative WS/DGAT belonging to marine OM60 clade. In contrast, soils samples contained phylotypes only related to actinomycetes. Among them, phylotypes affiliated with representatives largely or recently reported as oleaginous bacteria, as well as with others considered as possible lipid-accumulating bacteria based on the analysis of their annotated genomes. Our study shows for the first time that the environment could contain a higher variety of *ws/dgat* than that reported from bacterial isolates. The results of this study highlight the relevance of the environment in a natural process such as the synthesis and accumulation of neutral lipids. Particularly, both marine sediments and soil may serve as a useful source for novel WS/DGAT with biotechnological interest.

**Electronic supplementary material:**

The online version of this article (doi:10.1186/s13568-015-0128-1) contains supplementary material, which is available to authorized users.

## Background

Triacylglycerols (TAG) and wax esters (WE) are neutral lipids usually found in animals, plants, yeast and bacteria as reserve material. Particularly, Gram positive actinobacteria belonging to *Rhodococcus*, *Mycobacterium*, *Nocardia* and *Streptomyces* genera are able to synthesize and accumulate large amounts of TAG (Olukoshi and Packter 1994; Alvarez et al. 1996, 1997; Daniel et al. 2004). On the other hand, Gram negative gammaproteobacteria, such as *Acinetobacter* and some genera of marine hydrocarbonoclastic bacteria, usually accumulate WE and minor amounts of TAG (Bredemeier et al. 2003; Kalscheuer and Steinbüchel 2003; Kalscheuer et al. 2007). Thus, neutral storage lipids could be considered as a common trait in taxonomically non related bacterial strains, although it was only studied in a small number of bacteria. These bacterial lipids attracted the interest of the industrial market, as components of lubricants, polishes and cosmetics, or as a source for next generation biofuels (Röttig and Steinbüchel 2013). The traditional isolation and pure cultivation approach of oleaginous bacteria allowed a thorough physiological study on the lipid accumulation process occurring in oleaginous bacteria (Kalscheuer and Steinbüchel 2003; Wältermann et al. 2007). However, whether there is a wider and unknown diversity of lipid-accumulating bacteria in different natural environments remains to be investigated. Furthermore, the potential offered by terrestrial and marine habitats for the isolation of new lipid-accumulating bacteria or novel enzymes involved in their metabolism have been poorly explored. Hundreds of pharmaceutical and industrial components have been obtained from the environment but the storage of neutral lipids has been out of scope regardless of its biotechnological relevance.

TAG biosynthesis in bacteria has been proposed to occur via the Kennedy pathway, which includes three sequential acylation reactions of glycerol-3-phosphate intermediate catalyzed by different acyltransferases. The third acylation step of the glycerol backbone for the de novo TAG synthesis is catalyzed by a diacylglycerol acyltransferase (DGAT) enzyme, which is only present in WE- and TAG-accumulating bacteria (Kalscheuer and Steinbüchel 2003). These bacterial enzymes possess simultaneously both DGAT and acyl-CoA-fatty alcohol acyltransferase (wax ester synthase, WS) activities. In general, bacterial WS/DGATs are promiscuous enzymes, which accept a broad diversity of fatty acyl-CoAs and fatty alcohols as substrates for TAG and WE biosynthesis (Stöveken and Steinbüchel 2008). Interestingly, WS/DGAT enzymes (or also called Atf) from bacteria represent a new class of TAG synthesizing family, which exhibit no extended sequence similarity to any known eukaryotic acyltransferase (Kalscheuer and Steinbüchel 2003; Wältermann et al. 2007). While only one or few WS/DGAT enzymes occur in Gram negative bacteria able to synthesize WE and/or TAG, a high redundancy of these enzymes occurs in most TAG-accumulating actinobacteria (Alvarez and Steinbüchel 2010). Functionally similar enzymes (Atf or WS/DGAT) are found in Gram positive actinobacteria and Gram negative gammaproteobacteria exhibiting amino acid similarities ranging from 22 to 69% compared to AtfA from *Acinetobacter baylyi* ADP1 (Röttig and Steinbüchel 2013). Despite this low amino acid identity, sequence comparison revealed a highly conserved heptapeptide motif HHxxxDG found in most bacterial WS/DGAT (or its deduced sequence) that is critical for the enzymatic activity. Other motifs such as PLW and ND, involved in the correct folding of the active site, are also conserved in previously characterized Atf sequences (Villa et al. 2014). Due to its high diversity, an environmental survey of the genes encoding putative WS/DGAT enzymes might allow a greater resolution for the discovery of new classes of WS/TAG-synthesizing acyltransferases with interesting biotechnological properties, in comparison to those already known from previous physiological studies. It is of interest in this field to characterize new environmental WS/DGAT enzymes from diverse natural ecosystems with different specificities for acyl- or alcohol substrates of different physical properties. In this context, we provide the first evidence related to *ws/dgat* genes retrieved directly from the environment. San Jorge Gulf is an important petroleum basin located in Patagonia (Argentina). Both, soil and marine environments of this region are characterized by their low levels of nitrogen (Peressutti et al. 2003; Paparazzo et al. 2010). During extraction and transportation, oil spills may occur in the environment, aggravating the innate low availability of nitrogen. This scenario may favor the synthesis and accumulation of storage lipids due to the imbalance in carbon:nitrogen relation (C:N) tipping the balance in favor of carbon, the backbone of neutral lipids. Thus, the ability to synthesize and accumulate TAG/WS as an energy reserve may be an advantage in such natural environments.

Studies of natural habitats analyzing their possible role as genetic reservoirs of neutral lipids accumulation genes have been ignored. The purpose of our study is to explore the potential offered by different ecosystems, such as seawater, marine sediments and soils for finding novel genes coding WS/DGAT enzymes different from those already characterized. From the results obtained, we aimed at analyzing whether there could be a specific *ws/dgat* phylogeny associated with the environment under study. Based on our findings using molecular methods based on polymerase chain reactions, future traditional methods of culturing new bacteria could be focused towards specific bacterial groups. In this study, we provide the first evidences of the possible WS/DGAT presence and variation in nature by using unique PCR primers specifically targeting this bacterial gene.

## Methods

### Site description and sample collection

Sandy marine sediments from Comodoro Rivadavia Port (CR) and Belvedere Beach (BB) (S 45°85′ WO 67°46′ and S 45°96′ WO 67°56′, respectively) were collected from the top layer during a low tide period. Surface seawater (2 L) from those sites was collected simultaneously at 200 m from the shore. Water samples were filtered sequentially through 5 and 0.22 μm. The resulting membrane filters (Millipore) and collected marine sediments were stored at −80°C until DNA extraction. Two soil samples with different content of hydrocarbons were collected in Las Heras (S 46°5′ WO 68°95′), one of the most important oil-producing areas in Patagonia. LHIMP was collected from an oil field and 300 m away from it, in a cleaner area, LH sample was taken. Samples from marine and terrestrial habitats in Patagonia were collected during November 2011 and October 2012, respectively.

### Total petroleum hydrocarbon and water content in soil samples

Oil contamination was assessed by visual examination in all samples. Also, a subsample from each of them was subjected to chemical extraction using the established Environmental Protection Agency (EPA) technique 418.1 for the determination of total petroleum hydrocarbons by infrared spectrometry. Natural moisture content was determined in both soil samples by calculating the ratio of the weight of water to the weight of solids in 500 g of each sample according to ASTM D2216-90.

### DNA extraction

Total genomic DNA was extracted in triplicate from 0.5 g of marine sediment or soil using a MP FastDNA kit for soil (Qbiogene, Carlsbad, CA, USA) according to the manufacturer protocol. DNA from seawater was extracted as described in Lanfranconi et al. (2010) with freezing–heating steps carried out at −80 and 65°C, respectively.

### Primer design

Degenerated primers TgsX-up (5′-CGCCCGCTSTGGGAGATG-3′) and TgsX-down (5′-CGGGCCSGSGACGTTSGA-3′) were designed to hybridize to internal sequences of *ws/dgat* genes, coding for previously characterized WS/DGAT enzymes, from both *Actinobacteria* and *γ*-*Proteobacteria*. To identify conserved regions in DGAT proteins from *Actinobacteria*, sequences of representative orthologous proteins from *Rhodococcus opacus* PD630 (ACY38590.1, ACY38591.1 and ACY38595.1), *Rhodococcus jostii* RHA1 (YP_702929.1 and YP_701572.1), *Mycobacterium tuberculosis* H37Rv (NP_216276.1 and NP_215941.1), *Mycobacterium smegmatis* mc^2^ 155 (YP_890540.1 and YP_884705.1), *Streptomyces coelicolor* A3 (CAB61924.1) and *Nocardia farcinica* IFM 10152 (BAD56011.1 and BAD56419.1) were subjected to multiple alignment by clustalW (Larkin et al. 2007) (Additional file 1: Fig. S1a). The same analysis was carried out using sequences of orthologous proteins from *γ*-*Proteobacteria*, such as *Acinetobacter baylyi* ADP1 (AAO17391.1), *Alcanivorax borkumensis* SK2 (CAL18190.1 and CAL17252.1) and *Marinobacter hydrocarbonoclasticus* ATCC 49840 (YP_005430893.1, YP_005428090.1, ABO21022.1, YP_005431168.1, YP_005431092.1 and YP_005428634.1) (Additional file 1: Fig. S1b). Conserved regions occurring in most WS/DGAT proteins of both phyla, were selected to design the above mentioned primers (RPLWEM and SNVP/AGP for TgsX-up and TgsX-down, respectively). The codon usage of *A. baylyi* and *R. opacus* was considered to reverse translate the aminoacid sequences. Using the primer pair TgsX-up and TgsX-down, products of ~800 bp are expected to be amplified from typical WS/DGAT coding sequences.

Primers RhodoF (5′-GCAYCCSATGCACGTGGG-3′) and RhodoR (5′-ATCACGTTGAACGGCGG-3′) were designed to hybridize *ws/dgat* sequences from *Rhodococcus* genus. Sequences from *Rhodococcus opacus* PD630 (CP003949.1), *Rhodococcus jostii* RHA1 (YP_701572.1) and *Rhodococcus fascians* F7 (Rapid Annotation Subsystems Technology, RAST) isolated from petroleum contaminated soil in Patagonia were aligned by ClustalW (Additional file 1: Fig. S1c). RhodoF and RhodoR correspond to annealing positions 19-HPMHV-23 and 374-PPFNV-378, respectively of *R. opacus* PD630 enzyme, amplifying a ~1,000 bp product from *R. opacusws/dgat* gene. Primers AAP-F (5′-CARCCYATGCAYGTWGG-3′) and AAP-R (5′-ATMAYCASRTTGAAGGC-3′) were designed to hybridize *ws/dgat* sequences from different Gram negative bacteria. Sequences used to select specific regions included *Acinetobacter* sp. ADP1 (YP_045555.1), *Alcanivorax borkumensis* (WP_011590012.1) and *Psychrobacter cryhalolentis* (WP_011512619.1). Sequence alignment is shown in Additional file 1: Fig. S1d. Primer forward and reverse correspond to annealing positions 19-QPMHV-23 and 368-AFNLV-372, respectively of *Acinetobacter* sp. ADP1 WS/DGAT, amplifying a ~1,000 bp product from *Acinetobacterws/dgat* gene. Primers to amplify *ws/dgat* sequences from *Marinobacter hydrocarbonoclasticus* were Maratf1 (5′ACGCCCCTGAATCCCACT3′) and 130F (5′TGTCATGTTCAGGGCCAGYC3′). Sequences used to select specific regions included *Marinobacter aquaeolei* VT8 (CP000514) that is proposed as a synonym for *Marinobacter hydrocarbonoclasticus* (Márquez and Ventosa 2005) and *Marinobacter hydrocarbonoclasticus* ATCC49840 (FO203363). Primers correspond to annealing positions 2-TPLNPT-7 and 408-LALNMT-413, respectively of *Marinobacter aquaeolei* VT8, giving a fragment of 1,235 bp as product in PCR reactions.

### PCR amplification from environmental samples and cloning

Cycling parameters for PCRs using degenerated primers are shown in Table 1. Partial 16S rDNAs (~640 bp) were amplified using actinomycete-specific primers Act-0235 (5′CGCGGCCTATCAGCTTGTTG3′) and Act-0878 (5′CCGTACTCCCCAGGCGGGG3′) under previously established conditions (Stach et al. 2003). Reactions were done in a Mastercycler personal thermal cycler (Eppendorf) and contained 25 µl of a mixture including 10 mM Tris–HCl (pH 8.3), 2 mM MgCl2, 1.25% (v/v) DMSO, each dNTP at a concentration of 200 µM (InbioHighway, Argentina), 25 pmol (Act-0235 and Act-0878) or 50 pmol (TgsXup and TgsXdown) of each primer, 1.5 µl of template DNA and 1.25 U of Taq DNA polymerase (GoTaq, Promega, USA). To minimize PCR bias, reactions were carried out in duplicates and subsequently pooled. Freshly amplified PCR products were cloned into plasmid pGEM-T vector (Promega) according to commercial instructions. Six libraries were generated, four for soil samples (actinobacterial 16S rDNA and *ws/dgat*) plus two libraries for marine environments showing PCR amplification of the gene encoding WS/DGAT. Clone libraries from marine sediments were labelled with capital letters “CR” or “BB” for Comodoro Rivadavia Port and Belvedere beach, respectively. Those libraries from soil collected from Las Heras were designated with the capital letters “LH” or “LHIMP” according to the level of petroleum hydrocarbons found in the sampled sites. The label was followed by a number indicating the clone and the letter “l” in clones belonging to *ws/dgat* libraries.

**Table 1:** Primer pairs to amplify genes encoding WS/DGAT used in this study

Primer	Sequence (5′-3′)	Cycling conditions (°C s^−1^)	Specificity
TgsXup	CGCCCGCTSTGGGAGATG	94/300, (94/30, 58/30,72/60) ×30, 72/300	*Actinobacteria*/
TgsXdown	CGGGCCSGSGACGTTSGA		*γ*-*Proteobacteria*
Gram—F	CARCCYATGCAYGTWGG	94/300, (94/30, 45/45, 72/45) ×30, 72/300	Gram negative bacteria
Gram—R	ATMAYCASRTTGAAGGC		
RhodoF	CCGATGTGGGARMTGCAC	94/300, (94/30, 48/45, 72/45) ×30, 72/300	*Rhodococcus*
RhodoR	ATSATCACGTTGAACGGC		
Maratf1	ACGCCCCTGAATCCCACT	94/500, (94/60, 54/60, 72/90) ×30, 72/600	*Marinobacter*
Mar130F	TGTCATGTTCAGGGCCAGYC		

### Sequencing and phylogenetic analysis

Cloned inserts were amplified from selected clones by using M13 vector primers in PCR analysis and those products not producing the expected sizes were discarded. All clones from soil *ws/dgat* libraries were directly sequenced while a preliminary screening of diversity was done by restriction fragment length polymorphism (RFLP) with restriction endonuclease HhaI for 16S rDNA (New England Biolabs). Clones exhibiting the same RFLP pattern were grouped and representatives from every pattern, including those with only one representative, were sequenced. A similar approach was carried out for marine sediment *ws/dgat* libraries by digesting with restriction endonuclease HincII. Products to be sequenced were previously purified using Wizard SV Gel and PCR Clean-Up System (Promega). Sequences were obtained using the BigDye terminator cycle sequencing kit v3.1 (Applied Biosystems) according to the manufacturer’s instructions, and electrophoretic separation in a 3130xl and 3500xl Genetic Analyzers (Applied Biosystems) operated by the Genomic Unit of Biotechnology Institute in INTA Castelar, Argentina. The generated sequences were edited with BioEdit Sequence Alignment Editor v 7.0.9 (Hall 1999). After discarding the fragments corresponding to the primer pair used for the amplification of *ws/dgat*, clone sequences were translated and compared with reference sequences in databases using the blastp program (Altschul et al. 1990). Neighbour-joining, with Kimura approximation for calculation of distance matrices, and Parsimony treeing methods for phylogenetic analysis were done using the package Phylip (Felsenstein 1989). In case of 16S rDNA sequences, the program DECIPHER (Wright et al. 2012) was used to screen potential chimeras. Primer fragments were discarded and resulting sequences were compared to EBI database using blastn. Most similar reference sequences together with our environmental sequences were aligned using BioEdit Sequence Alignment Editor v 7.0.9 (Hall 1999). Neighbour-joining, with Jukes–Cantor approximation for calculation of distance matrices, and Parsimony treeing methods for phylogenetic analysis were done using the package Phylip (Felsenstein 1989). Bootstrap resampling of the NJ and P trees (1,000 and 100 replicates, respectively) was performed to provide statistical confidence to the topologies inferred. Assignation of sequences to sequence types was done with the program Mothur based on calculated distance matrices (Schloss et al. 2009). Only a representative from each sequence type was included in the phylogenetic trees.

### Nucleotide sequence accession numbers

Gene sequences generated in this study were deposited in GenBank and the accession numbers are KM095047 to KM095090 (16S rDNA) and KM114009 to KM114050 (*ws/dgat*).

## Results

### Environmental characteristics from sampled sites

Two non-vegetated soils of Las Heras, one from a contaminated oil extraction field (LHIMP) and another from a nearby area (LH) were collected simultaneously. Both samples presented low water content (3.5 and 3.8%, respectively), similar particle size of sand silt and clay and different values of total petroleum hydrocarbons (TPH). The presence of hydrocarbons was visually clear in LHIMP sample, with a content of 48.5 g kg^−1^ TPH as revealed by chemical analysis. Oil was not visually detected in LH sample but the chemical analysis indicated the presence of low concentrations of hydrocarbons (0.2 g kg^−1^ TPH).

Additionally, samples were collected from marine ecosystems (sediments and seawater) in the main port of Comodoro Rivadavia city and in Belvedere beach located in a deserted not urbanized area in Chubut Province, Patagonia, Argentina. In all marine samples the content of TPH was below the detection limit of the used technique.

### Testing of primers with pure cultures

Primer validation was performed using DNA from bacterial strains including *Rhodococcus jostii* RHA1, *Rhodococcus opacus* PD630, *Streptomyces coelicolor* A3 (2), *Nocardia asteroides* 419, *Mycobacterium ratisbonense* SD4. All of them yielded amplicons of the predicted size using primers TgsXup/TgsXdown. In contrast, other bacterial strains such as *Amycolatopsis tucumanensis*, *Dietzia* sp. A12, *Gordonia amicalis* 713, *Marinobacter hydrocarbonoclasticus* DSM8798 and *Acinetobacter baylyi* ADP1 did not give an amplification product. AAP-F/AAP-R primers were also validated in PCR reactions using DNA from *Acinetobacter baylyi* ADP1 that showed an amplification product, while *Marinobacter hydrocarbonoclasticus*, as expected, showed no amplification. The other primers used in this study, RhodoF/RhodoR and Maratf1/Mar130F, were tested in PCR analysis using DNA from *Rhodococcus fascians* F7, *Rhodococcus opacus* PD630 and *Rhodococcus jostii* RHA1 or *Marinobacter hydrocarbonoclasticus* DSM8798 as templates, respectively. A fragment with the expected size was obtained in all analyzed strains.

### WS/DGAT genes in soil samples

**Fig. 1:**
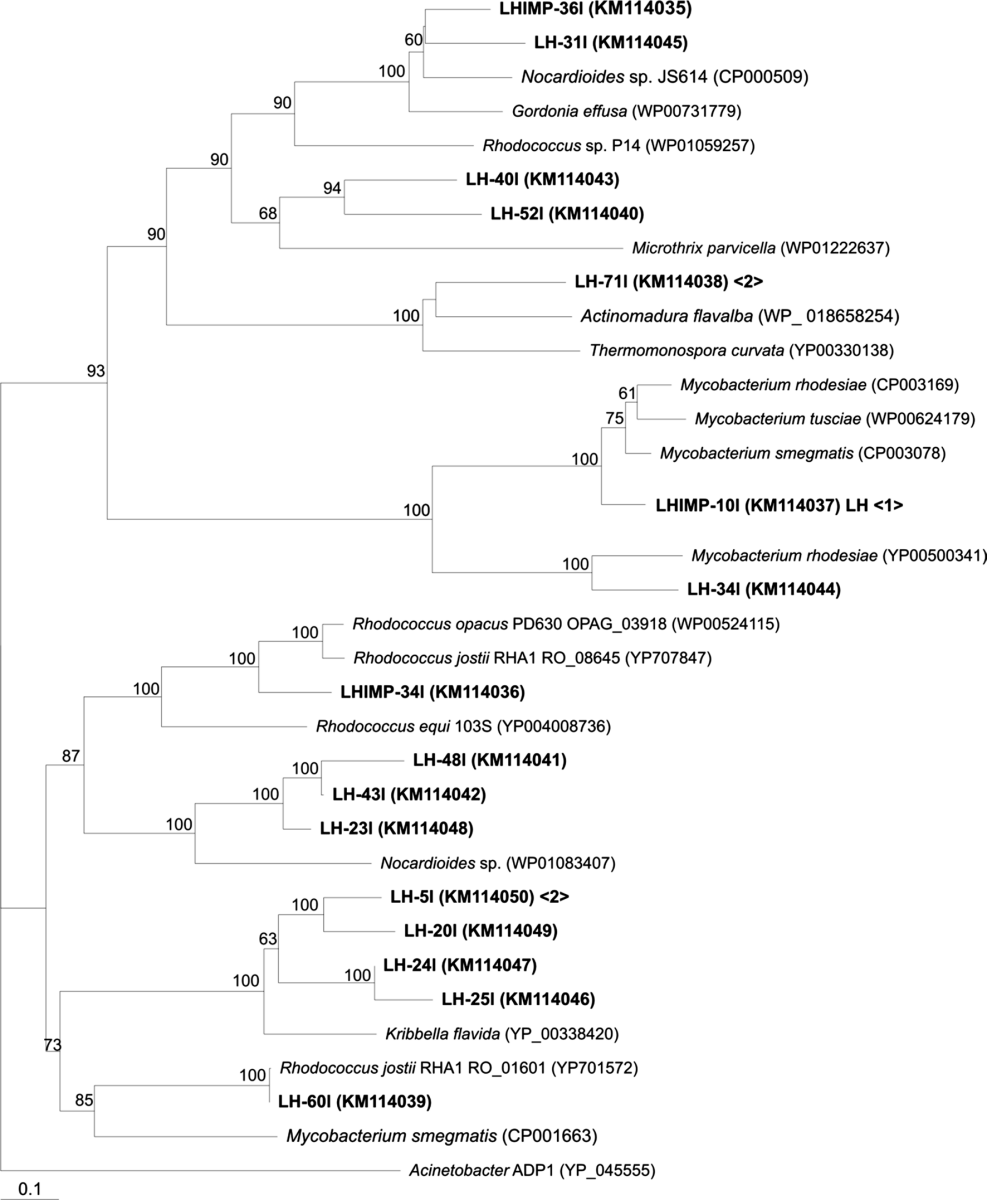
Neighbour-joining dendogram showing the relationship of translated *ws/dgat* sequences (272 amino acids positions) obtained from Patagonian soils with reference sequences. The tree was calculated with a representative of each sequence type defined at 0.01 distance cut-off. For environmental sequence types, *numbers in parenthesis* indicate the accession numbers given in GenBank. Total number of sequences belonging to the same sequence type in the corresponding library from which they were retrieved is indicated in < and > symbols. AtfA from *Acinetobacter baylyi* ADP1 was used as outgroup. Relevant bootstrap values (1,000 replicates) higher than 50% are shown.

Conserved sequences of genes coding for putative WS/DGAT enzymes were PCR amplified using primers TgsXup and TgsXdown (Table 1; Additional file 1: Fig. S1) and DNA from soil samples collected in Patagonia. This area, located in South Argentina, is characterized by its extreme temperatures, semiarid conditions and high petroleum extraction activity. Orthologs of *ws/dgat* were successfully amplified from two soil samples in Patagonia, one highly polluted with hydrocarbons (LHIMP) and the other without environmentally relevant hydrocarbon levels (LH). Interestingly, all sequences recovered affiliated with genes from Gram-positive actinomycetes (Fig. 1). Abundance of *ws/dgat* sequences was low in both soil samples, with LHIMP sequences falling in three clusters while sequences belonging to LH library were dispersed in the phylogenetic tree with a total of seven clusters (Fig. 1). There was a strong composition overlap between soil libraries with two out of three clusters from LHIMP library containing sequences also found in LH library (Fig. 1). For example, clone LHIMP-36 l clustered together with clone LH-31l and WS/DGAT sequences from *Rhodococcus* sp. P14 (Song et al. 2011), *Gordonia effusa* and *Nocardiodes* sp. JS61 (Coleman et al. 2011).

Most sequences amplified from LH soil sample fall into clusters containing WS/DGAT sequences of bacteria recently considered as “possible oleaginous” based on the information of their complete genome sequencing. Among these strains, *Microthrix parvicella* strain RN1 was isolated from wastewater (McIlroy et al. 2013). On the other hand, several LH clone sequences clustered with a WS/DGAT sequence of *Kribbella flavida*, related to the Nocardioidaceae family (Fig. 1), in which its type strain was isolated from a soil sample in Korea (Pukall et al. 2010). Other LH clones clustered with sequences of *Nocardioides* strains (Fig. 1). Other cluster, represented by clone LH-71l together with WS/DGAT enzymes from *Actinomadura flavalba* and *Thermomonospora curvata*, was found in phylogenetic analyses of our libraries. Interestingly, an amplified sequence from LH soil sample was closely related to RO_01601 gene from *Rhodococcus jostii* RHA1, which is the orthologous counterpart of *atf*2 in *R. opacus* PD630 (Fig. 1). This gene coding for a WS/DGAT enzyme involved in TAG biosynthesis and accumulation in strain PD630 has been previously characterized in detail (Hernández et al. 2013). To confirm the results obtained with TgsX primers, PCR analysis of both soil samples were performed with two specific primer pairs to amplify WS/DGAT from *Rhodococcus* and Gram negative bacteria (Table 1). Although weak, a positive PCR product of the expected size was obtained with RhodoFxRhodoR. In contrast, no amplification was observed when AAP-FxAAP-R was used in the reaction (data not shown).

Taken together, all amplified sequences from both soil samples clustered with putative WS/DGAT genes from actinomycetes. Since some members of *Actinobacteria* can be considered as specialists in TAG accumulation, and to provide further information on diversity of this relevant group in neutral lipid storage, we performed an analysis of actinobacterial 16S rRNA diversity in those samples showing the presence of WS/DGAT genes affiliated with this family (Additional file 1: Fig. S2). In addition, the obtained results may also be useful to confirm the coverage of TgsX primers for amplification of WS/DGAT from the environment. With the exception of clones related to *Cellulomonas* in LH soil sample, most sequences found represented genera considered as lipid-accumulating *Actinobacteria*. Among them, a high number of clones in polluted soil affiliated with *Dietzia* (Additional file 1: Fig. S2). Our clones were closely related to *Dietzia* sp. H0B, a strain isolated from a polluted area after Prestige oil spill and able to degrade diverse alkanes from C_12_ to C_38_ (Alonso-Gutierrez et al. 2011). *Streptomyces*, a genus previously reported for its capacity for accumulating TAG (Olukoshi and Packter 1994; Kaddor et al. 2009), was present in both, LH and LHIMP libraries. In spite of including *Streptomyces* WS/DGAT sequence for TgsX800 primers design (Additional file 1: Fig. S1a) and its positive amplification from a pure culture of *Streptomycescoelicolor*; no sequences affiliated to this genus were detected in our environmental PCR analyses covering *ws/dgat*. According to the analysis of complete genomes available in public databases, the ability to accumulate TAG seems to occur in some but not all species of this genus. Thus, we could have retrieved 16S rDNA sequences from those representatives with no *ws/dgat* genes.

Several phylotypes affiliated with the genera *Nocardioides,Kribbella, Actinomadura,* and *Thermomonospora* were retrieved mostly from LH soil sample (Additional file 1: Fig. S2). This result correlated well with those obtained after PCR amplification of *ws/dgat* sequences from LH soil sample (Fig. 1; Additional file 1: Fig. S2).

Interestingly, sequences closely related to *Rhodococcus* genus, especially to *R. fascians*, dominated the 16S rDNA library constructed from LHIMP soil sample in this study (Additional file 1: Fig. S2). These microorganisms were frequently isolated from polluted soils in Patagonia (Peressutti et al. 2003; Bequer Urbano et al. 2014) and in other places of the world (Seto et al. 1995; Alvarez et al. 1996; Na et al. 2005). In this context, some of our 16S rRNA clones, principally from LH soil sample, were affiliated with uncultured clones retrieved from hyper-arid environments such as Dry Valleys in Antarctica or Atacama Desert (Additional file 1: Fig. S2).

### WS/DGAT genes in marine samples

**Fig. 2:**
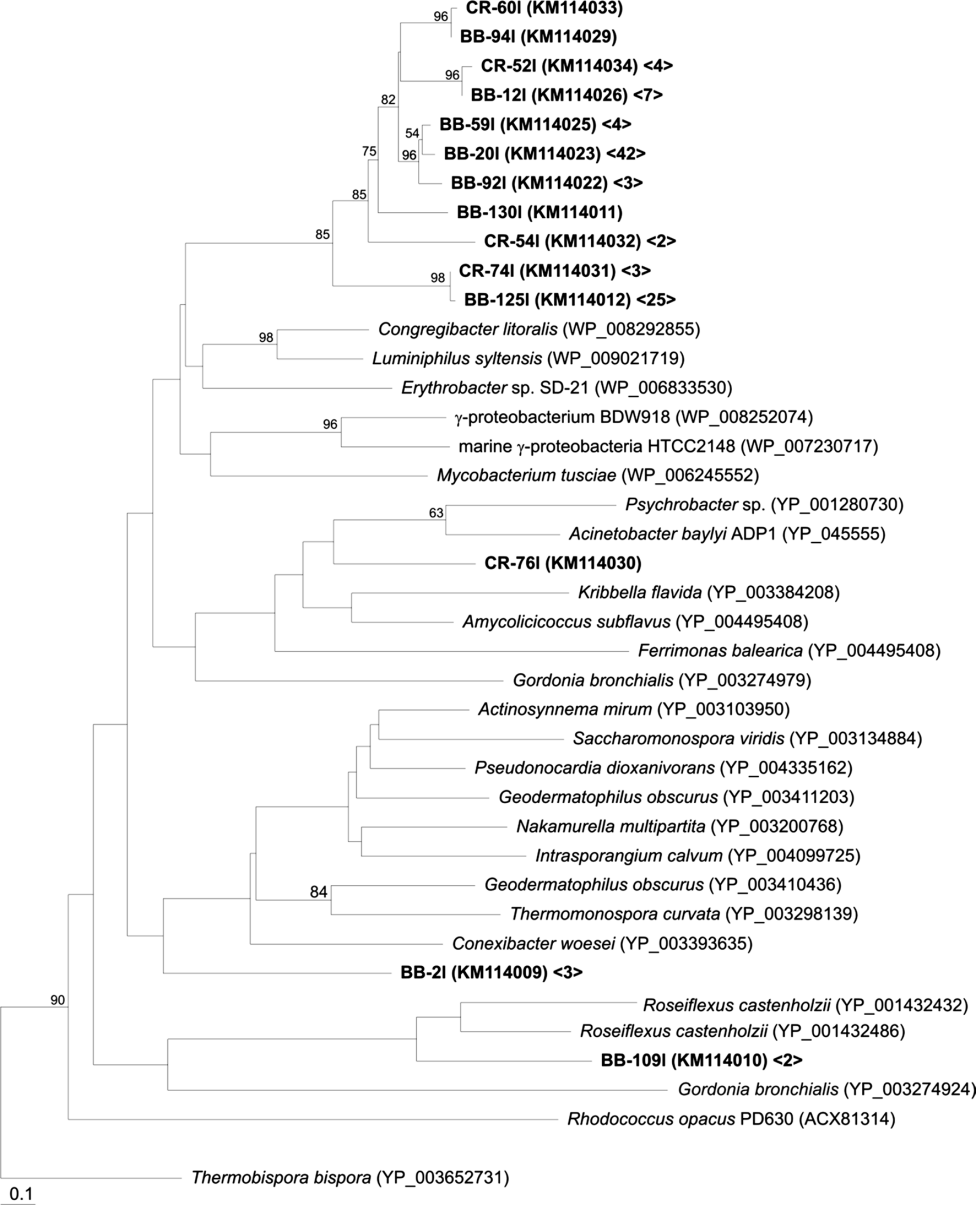
Neighbour-joining dendogram showing the relationship of translated *ws/dgat* sequences (278 amino acids positions) obtained from Patagonian marine sediments with reference sequences. The tree was calculated with a representative of each sequence type defined at 0.01 distance cut-off. For environmental sequence types, numbers in < and > symbols indicate the total number of sequences in the corresponding library from which they were retrieved. The accession numbers of reference sequences and environmental clones obtained in this study are indicated. Acyltransferase from *Thermobispora bispora* was used as outgroup. Relevant bootstrap values (1,000 replicates) higher than 50% are shown.

Marine DNA-extracts were screened by PCR to analyze the diversity of bacteria potentially able to synthesize and accumulate WE and/or TAG present in sediment samples collected from Comodoro Rivadavia Port (CR) and Belvedere Beach (BB). Most environmental sequences clustered together in a unique group with no cultured or uncultured representatives retrieved from databases (Fig. 2). Within this cluster, there were sequence types belonging to both sampled sites showing 50–98% sequence similarities among them. The closest cultured strains containing putative WS/DGAT were *Congregibacter litoralis* and *Luminiphilus syltensis* both belonging to OM60/NOR5 clade of marine gammaproteobacterial isolates from coastal seawater (Spring et al. 2013). Proteins with high identity values (59%) to the sequences retrieved in our study were recently annotated as WS/DGAT enzymes in these strains. Due to the recent release of the genome annotation in these bacteria, no reports about the ability of such microorganisms to accumulate WE or TAG have been reported so far. This situation, where proteins are firstly annotated with its putative function, which is later confirmed, is usually found. Recent examples include *Thermomonospora curvata* (Moncalián et al. 2014) and *Microthrix parvicella* (McIlroy et al. 2013).

**Fig. 3:**
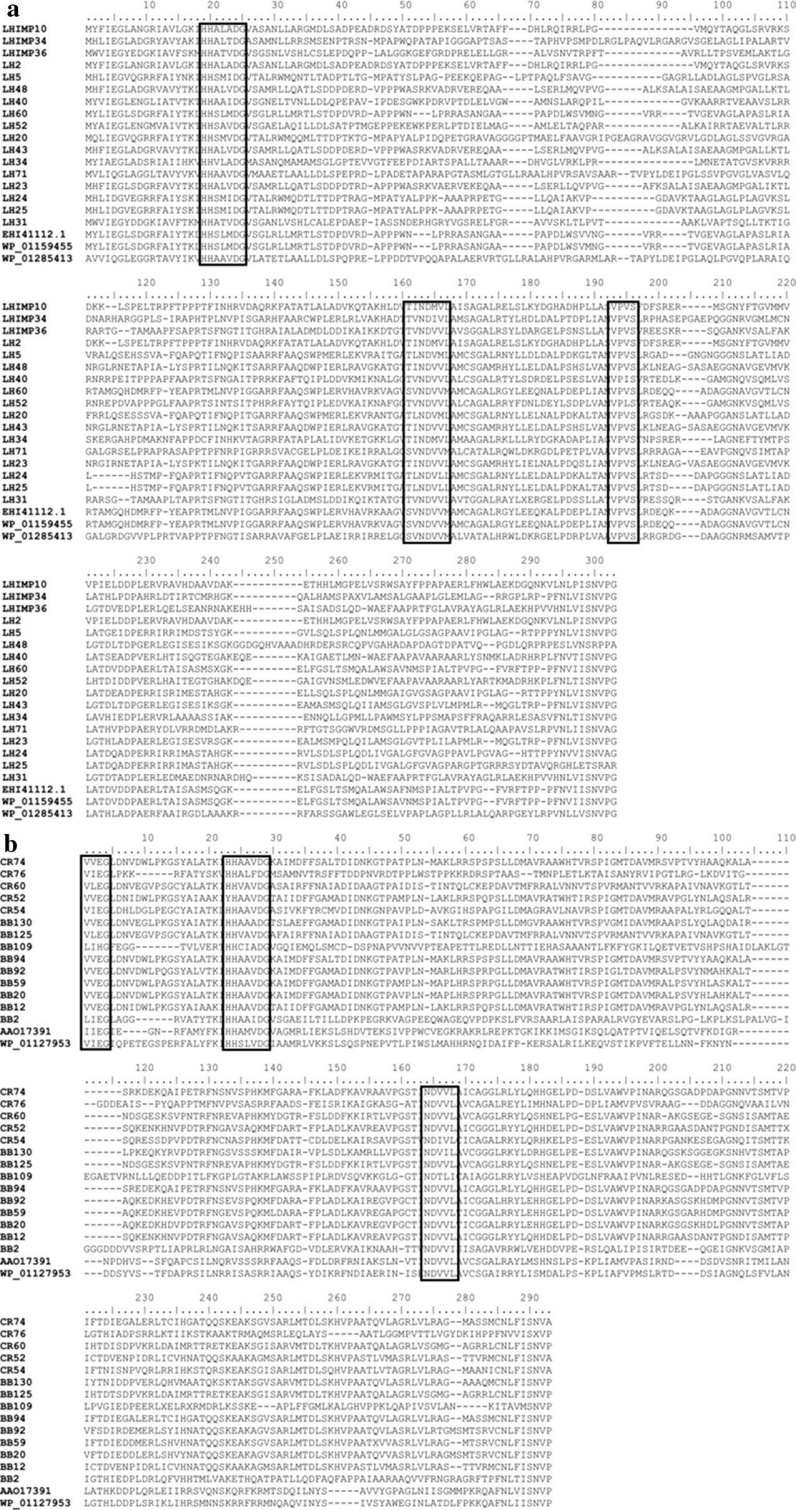
Multiple sequence alignments of Patagonian environmental clones and characterized WS/DGAT. Conserved regions and domains required for acyltransferase activity are marked in *boxes*. **a** LHIMP and LH = Las Heras soil more or less impacted, respectively. Atf-like acyltransferases from *Rhodococcus opacus* PD630, *Rhodococcus jostii* RHA1 and *Thermomonospora curvata* are included. **b** CR and BB = Comodoro Rivadavia port and Belvedere beach sediments, respectively. Atf from *Acinetobacter baylyi* ADP1 and Atf-like from *Psychrobacter articus* 273-4 are included.

The amplified sequence of the clone CR-76l retrieved from the Port sample shared a 39% identity with the well characterized AtfA from *Acinetobacter baylyi* ADP1 and a 40% with a putative WS/DGAT from *Psychrobacter* sp. PRwf-1. Both genera contain characterized Atf-like acyltransferases (reviewed in Röttig and Steinbüchel 2013). AtfA from strain ADP1 is a promiscuous enzyme able to accept a broad variety of substrates for the production of diverse WE (Kalscheuer and Steinbüchel 2003). This enzyme has been used for the production of biodiesel (microdiesel) after heterologous expression in *Escherichia coli* (Kalscheuer et al. 2006); thus, it is considered as a relevant tool for engineering new microbial biofuels. The increased number of available complete genomes from bacteria has produced an enormous amount of new sequences annotated as hypothetical proteins, which contain the conserved regions of WS/DGAT enzymes. Among them, OPAG_03918 from *Rhodococcus opacus* PD630 annotated as hypothetical protein is commonly considered as a WS/DGAT (Hernández et al. 2013). Another example is found in *Roseiflexus castenholzii*, a Gram negative and thermophilic bacterium isolated from a bacterial mat (Hanada et al. 2002). Clones from BB sample, represented by BB-109l, showed the highest identity with a hypothetical protein containing the domains that define it as WS/DGAT from this strain. Furthermore, we found clones distantly related to actinobacterial WS/DGAT enzymes (clone BB-2l) also in BB sample. In addition, the other primer pairs presented in Table 1 were used in PCR analyses. No amplification was detected with the other tested pairs that included RhodoF/RhodoR, AAP-F/AAP-R and Maratf1/Mar130F primer pairs all targeting WS/DGAT. We also analyzed the occurrence of WS/DGAT genes in surface seawater, but no amplification was obtained in our samples with any of the primers tested. Most of the amplified marine sequences of this study exhibited low identities with known WS/DGAT enzymes and showed no close relatives, suggesting the phylogenetic diversity and novelty of these genes. 3D modeling of WS/DGAT indicates that these enzymes contain the conserved HHxxxDG peptide as well as other conserved residues such as ^118^PLW^120^ and ^270^ND^271^ that characterize WS/DGAT enzymes (Villa et al. 2014). Our environmental sequences possess the highly conserved HHxxxDG and also the ND motifs with identical sequence to the WS/DGAT (Fig. 3). The PLW domain was not considered in our analysis because this sequence domain was part of TgsXdown primer. Apart from specific domains, the low sequence identity between WS/DGAT and other CoA-dependent acyltransferases family make sequence comparison a good approach to differentiate them. Our sequences showed the highest homology to sequences from databases annotated as WS/DGAT. Furthermore, primers to amplify WS/DGAT genes were specifically designed from genes coding characterized WS/DGAT enzymes from both, Gram negative and Gram positive bacteria (Additional file 1: Fig. S1a, b).

## Discussion

In this study, an important number of genes coding putative new WS/DGAT enzymes were obtained from environmental samples collected in different natural habitats. Several of the representatives obtained affiliated to WS/DGAT from different genera in which the ability to accumulate neutral lipids has never been reported. Marine sediments were collected from Comodoro Rivadavia Port, an area with a long history of hydrocarbon presence (Commendatore et al. 2000; Esteves and Commendatore 1993; Nievas et al. 2012). Surprisingly, no TPH were detected in CR sample and *ws/dgat* sequences obtained were similar to those found in BB, an area considered as pristine or no impacted by human activities. Based on the sequences retrieved from these areas, marine sediments could be considered as a valuable source of bacterial WS/DGAT enzymes with biotechnological relevance. Most sequences were related to strains affiliated to OM60 group, belonging to *γ*-*Proteobacteria*. Unfortunately, we could not confirm the results obtained with TgsX primers using AAP primers. These latter were designed before knowing our results on enzyme diversity; thus, the sequences from OM60 clade were not included in the alignment used to construct the primers. *In silico* testing of sequence hybridization indicated the presence of mismatches that could result in our finding of negative PCR amplification. No sequences affiliated with *Actinobacteria* were found in marine sediments. However, clones distantly related to *ws/dgat* genes belonging to this phylum were found in BB sample. These clones could belong to genes coding new WS/DGAT from uncultured bacteria.

According to our results and considering the lack of reports showing the capacity of lipid storage in pelagic marine bacteria thriving in unaffected habitats, we hypothesize that the environmental conditions found in upper water column might not favor the presence of bacteria with the genetic potential to accumulate TAG or WE. Marine hydrocarbonoclastic bacteria (HCB), which are able to accumulate TAG and/or WE (Holtzapple and Schmidt-Dannert 2007; Manilla-Pérez et al. 2011), are usually found in polluted waters 4 weeks after an acute oil spill (Kostka et al. 2011). This early response to the contamination declines after a few weeks (reviewed in Head et al. 2006). Considering the short-time response of HCB during an oil spill and the absence of TPH in our marine samples, finding WS/DGAT affiliated with this group was not expected in the analyzed ecosystems. Even though the sequences retrieved from CR and BB affiliate with WS/DGAT from *γ*-*Proteobacteria*, none of them were related to HCB. The results of this study demonstrated that marine environments may serve as a source of new collections of bacterial WS/DGAT genes.

Data related to the occurrence of TAG-accumulation in soil environments of Patagonia (Argentina) has been previously reported (Alvarez 2003; Silva et al. 2010; Bequer Urbano et al. 2014). However, all these studies were focused on bacterial isolates. Thus, to our knowledge this is the first report on the analysis of *ws/dgat* gene in soil environments. Our findings not only correlate with those obtained from culturing methods, but also broaden the diversity of genes involved in the process. All sequences recovered from soil samples affiliated with actinobacterial *ws/dgat* genes. This result may indicate the ability of such microorganisms to adapt and survive in semiarid soil environments beyond the level of hydrocarbon impact. Few sequences were retrieved from polluted soil, all of them related to putative *ws/dgat* from strains isolated from contaminated sediment or soil with the capacity to degrade hydrocarbons (Coleman et al. 2011; Song et al. 2011). The capability for accumulating lipids by these bacterial strains belonging to *Nocardioides* and *Rhodococcus* genera was not analyzed; however, the biosynthesis and accumulation of TAG is a known feature of bacteria belonging to *Rhodococcus* genus (Alvarez and Steinbüchel 2010). Other clones of this study clustered with sequences from TAG-accumulating actinobacteria different to *Rhodococcus*. In this context, sequences from both LHIMP and LH libraries affiliated with reference sequences of non-pathogenic saprophytic *Mycobacterium* sp. strains. Bacteria belonging to this genus have environmental and pathogenic representatives with the ability to synthesize TAG and/or WE according to the available substrate and under diverse stress conditions (Sirakova et al. 2006; Silva et al. 2007).

Our environmental survey showed that most sequences from LH soil sample were affiliated to WS/DGAT sequences of bacteria recently considered as “possible oleaginous” such as *Microthrix parvicella* strain RN1, which exhibited a high redundancy of *ws/dgat* sequences, containing at least 10 putative genes (McIlroy et al. 2013). Its ability to accumulate TAG was suggested after electronic microscopy analysis showing transparent inclusions and the lack of a genetic capacity for polyhydroxyalkanoates biosynthesis. A high similarity was observed for LH clone sequences clustering with a WS/DGAT sequence of *Kribbella flavida* (Pukall et al. 2010). To our knowledge there are no previous reports on the occurrence of bacteria belonging to *Kribbella* or *Microthrix* genera in natural environments of Patagonia. Furthermore, there are no studies focused on TAG accumulation by these microorganisms, except a recent in silico study based on the metabolic pathway involved in neutral lipid accumulation in *M. parvicella* Bio17-1 (Muller et al. 2014). Other LH clones clustered with sequences of *Nocardioides* strains (Fig. 1). *Nocardioides* members are frequently isolated from hydrocarbon-contaminated soil samples, showing the ability to degrade diverse *n*-alkanes (C_2_ to C_16_) (Kimbrel et al. 2013). In spite of its potential applications in bioremediation and ecological relevance, no in-depth genome analysis has been performed yet in such microorganisms. WS/DGAT from *Thermomonospora curvata* was also represented in LH library. A patent based on the efficiency of WS/DGAT from *Thermomonospora curvata* in the production of TAG has been recently published (Moncalián et al. 2014). The authors claimed that the heterologous expression of this gene in *Escherichia coli* showed the highest levels of TAG accumulation in comparison with other cloned representatives from *Acinetobacterbaylyi* ADP1 (Stöveken et al. 2005), *Marinobacter aquaolei* (Holtzapple and Schmidt-Dannert 2007) and *Alcanivorax borkumensis* (Kalscheuer et al. 2007). Finding representatives affiliated to WS/DGAT from *Thermomonospora curvata* may indicate that the objective of scanning the environment to obtain different WS/DGAT with novel properties is headed in the correct direction. Also, rhodococcal isolates from Patagonia able to produce variable amounts of TAG during cultivation on hydrocarbons and other carbon sources have largely been reported (Alvarez 2003; Silva et al. 2010; Bequer Urbano et al. 2014). The 16S rRNA sequences of rhodococci were well represented in the polluted soil sample analyzed in this study. These results underlay the important role of rhodococci in bioremediation of polluted soils and their successful adaptation to arid environments. Another interesting finding derived from 16S rDNA analysis includes *Dietzia*. An important number of LHIMP clones were affiliated with representatives of this genus. Within it, a strain of *D. maris* previously isolated from a soil sample of Patagonia (Argentina) (Pucci et al. 2000) was able to produce TAG when grown on hexadecane as sole carbon source under nitrogen-limiting conditions (Alvarez 2003). However, our primers did not cover the sequences of *ws/dgat* found in this genus. Altogether, semiarid soils of Patagonia (Argentina) seem to be enriched by actinomycetes with the ability to synthesize and accumulate TAG and/or WE and to tolerate arid conditions.

This work provides the first evidence of natural environments as a valuable source of novel bacterial *ws/dgat* genes. Natural ecosystems show a differential distribution of this enzyme, with actinobacteria occurring predominantly in soils and Gammaproteobacteria in marine sediments. The results obtained in this study confirmed that the selected primers were efficient and covered *ws/dgat* genes including those occurring in Gram positive as well as Gram negative bacteria. However, the presence of other *ws/dgat* genes could have been overlooked by the primers used.

The analysis of *ws/dgat* diversity by culture-independent methods may be useful to direct future environmental search for the isolation of bacterial strains with an enzymatic potential for biotechnological production of unusual lipids with new technological applications. In this context, our findings show group-specific *ws/dgat* according to the site under study and suggest that culturing efforts should be directed towards *γ*-*Proteobacteria* in marine environments. In contrast, the focus should be on *Actinobacteria* in terrestrial habitats.

## Additional file

Additional file 1:
**Fig. S1.** Multiple sequence alignment between ws/dgat sequences used to construct primers. (A) TgsX800up and down from Actinobacteria (B) TgsX800 from Proteobacteria (C) RhodoF and RhodoR (D) Gram-F and Gram-R. Conserved amino acids are displayed in black and homologous residues in grey. **Fig. S2.** Phylogenetic tree of 16S rDNA sequences affiliated to *Actinobacteria.* The tree was calculated with a representative of each sequence type defined at 0.03 distance cut-off. Numbers in < > indicate total clones retrieved from the corresponding library. The accession numbers for reference sequences and environmental clones obtained in this study are indicated in parenthesis. *Thermodesulfatator indicus* was used as outgroup. Relevant bootstrap values (1,000 replicates) higher than 50% are shown.
