# Supplementary material for: Identification of genes coding for putative wax ester synthase/diacylglycerol acyltransferase enzymes in terrestrial and marine environments
Source: AMB Express. 2015 Jul 31;5:42. doi: 10.1186/s13568-015-0128-1 (PMC4520822; doi:10.1186/s13568-015-0128-1)

**Additional Files**

**Article title:**

Identification of genes coding for putative wax ester synthase/diacylglycerol acyltransferase enzymes in terrestrial and marine environments.

**Journal name:**

AMB Express

**Authors:**

Lanfranconi MP, Alvarez AF, Alvarez HM.

**Affiliation and e-mail of corresponding author:**

Corresponding author: Héctor M. Alvarez, Centro Regional de Investigación y Desarrollo Científico Tecnológico (CRIDECIT), Facultad de Ciencias Naturales, Universidad Nacional de la Patagonia San Juan Bosco y CIT-CHUBUT CONICET, Km. 4- Ciudad Universitaria, 9000 Comodoro Rivadavia, Chubut, Argentina. Tel. +54 297 4522192/fax: +54 297 4550339; E-mail: [halvarez@unpata.edu.ar](mailto:halvarez@unpata.edu.ar).

**Supplementary Figure legends**

**Figure S1** Multiple sequence alignment between ws/dgat sequences used to construct primers (A) TgsX800up and down from Actinobacteria (B) TgsX800 from Proteobacteria (C) RhodoF and RhodoR (D) Gram-F and Gram-R. Conserved amino acids are displayed in black and homologous residues in grey.

**Figure S2** Phylogenetic tree of 16S rDNA sequences affiliated to *Actinobacteria*. The tree was calculated with a representative of each sequence type defined at 0.03 distance cut-off. Numbers in < > indicate total clones retrieved from the corresponding library. The accession numbers for reference sequences and environmental clones obtained in this study are indicated in parenthesis. *Thermodesulfatator indicus* was used as outgroup. Relevant bootstrap values (1000 replicates) higher than 50% are shown.

**Figure S1A**

**Figure S1B**

**Figure S1C**

**Figure S1D**

**Figure S2**


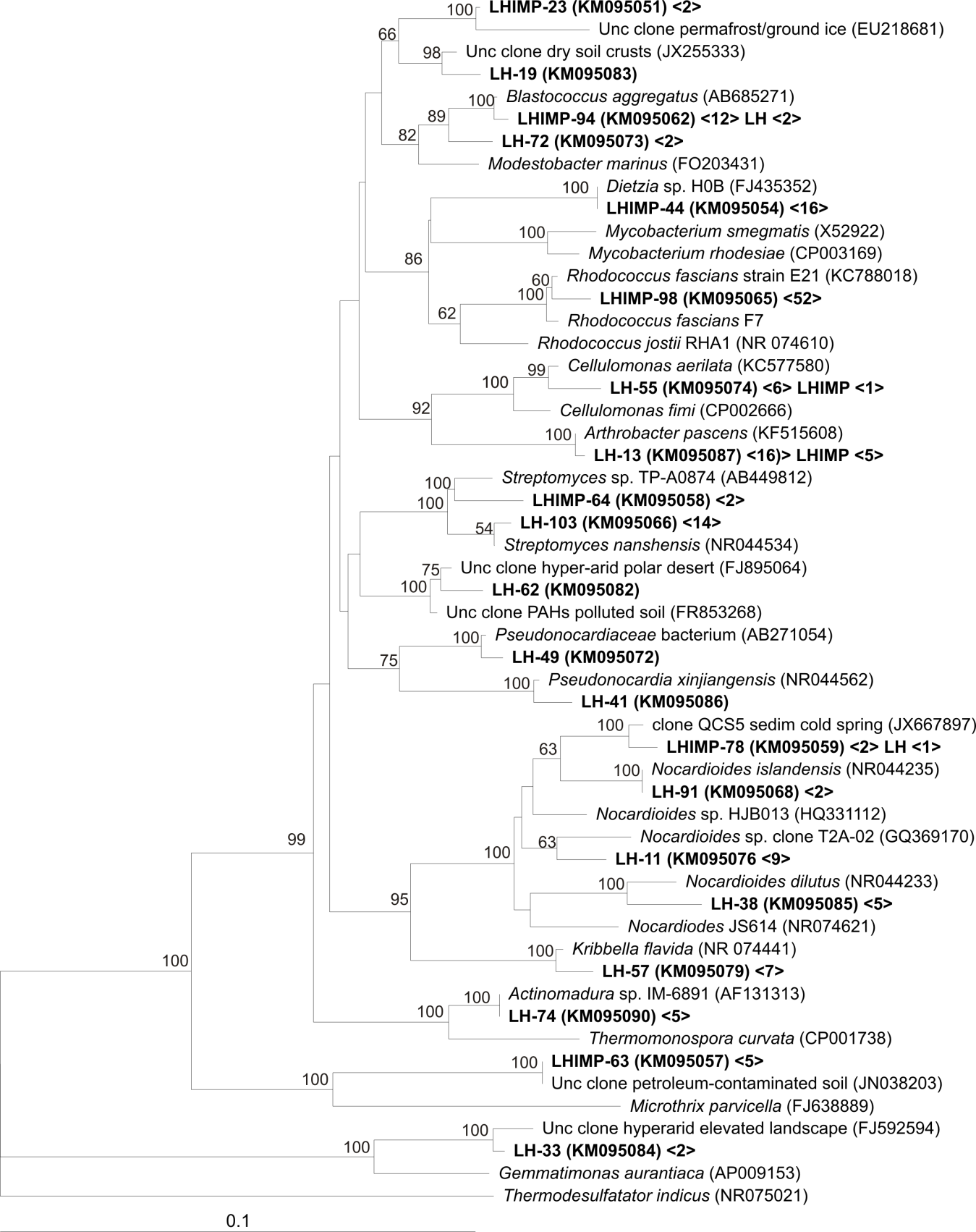

Supplement: Additional file 1: — Fig. S1. Multiple sequence alignment between ws/dgat sequences used to construct primers. (A) TgsX800up and down from Actinobacteria (B) TgsX800 from Proteobacteria (C) RhodoF and RhodoR (D) Gram-F and Gram-R. Conserved amino acids are displayed in black and homologous residues in grey. Fig. S2. Phylogenetic tree of 16S rDNA sequences affiliated to Actinobacteria. The tree was calculated with a representative of each sequence type defined at 0.03 distance cut-off. Numbers in < > indicate total clones retrieved from the corresponding library. The accession numbers for reference sequences and environmental clones obtained in this study are indicated in parenthesis. Thermodesulfatator indicus was used as outgroup. Relevant bootstrap values (1,000 replicates) higher than 50% are shown. [file 13568_2015_128_MOESM1_ESM.docx]
